# Obstructive Sleep Apnea-Associated Intermittent Hypoxia-Induced Immune Responses in Males, Pregnancies, and Offspring

**DOI:** 10.3390/ijms25031852

**Published:** 2024-02-03

**Authors:** Ruolin Song, Tracy L. Baker, Jyoti J. Watters, Sathish Kumar

**Affiliations:** 1 Department of Comparative Biosciences, School of Veterinary Medicine, University of Wisconsin, Madison, WI 53706, USA; ruolin.song@wisc.edu (R.S.); tracy.baker@wisc.edu (T.L.B.); jjwatters@wisc.edu (J.J.W.); 2 Department of Obstetrics and Gynecology, School of Medicine and Public Health, University of Wisconsin, Madison, WI 53792, USA

**Keywords:** obstructive sleep apnea, intermittent hypoxia, inflammation, male, pregnancy, offspring, cardiovascular system

## Abstract

Obstructive sleep apnea (OSA), a respiratory sleep disorder associated with cardiovascular diseases, is more prevalent in men. However, OSA occurrence in pregnant women rises to a level comparable to men during late gestation, creating persistent effects on both maternal and offspring health. The exact mechanisms behind OSA-induced cardiovascular diseases remain unclear, but inflammation and oxidative stress play a key role. Animal models using intermittent hypoxia (IH), a hallmark of OSA, reveal several pro-inflammatory signaling pathways at play in males, such as TLR4/MyD88/NF-κB/MAPK, miRNA/NLRP3, and COX signaling, along with shifts in immune cell populations and function. Limited evidence suggests similarities in pregnancies and offspring. In addition, suppressing these inflammatory molecules ameliorates IH-induced inflammation and tissue injury, providing new potential targets to treat OSA-associated cardiovascular diseases. This review will focus on the inflammatory mechanisms linking IH to cardiovascular dysfunction in males, pregnancies, and their offspring. The goal is to inspire further investigations into the understudied populations of pregnant females and their offspring, which ultimately uncover underlying mechanisms and therapeutic interventions for OSA-associated diseases.

## 1. Introduction

Obstructive sleep apnea (OSA) is a multifactorial respiratory disorder characterized by repeated episodes of airway collapse, resulting in intermittent hypoxemia, hypercapnia, and sleep fragmentation. OSA sex-specifically affects nearly 1 billion adults worldwide [1], with its occurrence ~2.5 times higher in men than in premenopausal women [2]. However, pregnancy increases the occurrence of OSA from 8.4% at baseline (first trimester) to 19.7% by the third trimester when adjusted for body mass index (BMI) [3], thus reaching a prevalence similar to men [4]. OSA has emerged as a public health burden because of its morbidities and associated complications [5], especially cardiovascular diseases such as systemic hypertension [6], stroke [7], and preeclampsia [8]. Notably, gestational OSA experienced by the mother has long-lasting deleterious consequences also for the offspring [9].

A typical pattern coupled with the majority of respiratory events during OSA involves repetitive, short cycles of desaturation followed by rapid reoxygenation [10]. Such a sequence of desaturation and reoxygenation exposes organs and tissues to episodic hypoxia and normoxia. Therefore, using intermittent hypoxia (IH) exposure to experimentally mimic the hypoxemic events experienced in OSA may provide valuable insights into OSA and its pathogenetic pathways. The exact mechanisms underlying OSA-induced cardiovascular disease remain elusive. However, inflammatory processes and oxidative stress are considered to play a key role [11,12].

This review will focus on the major effect of IH and its underlying inflammatory mechanisms linked to cardiovascular outcomes in males, pregnancies, and offspring. It aims to integrate the acquired knowledge on IH-induced inflammatory mechanisms in male subjects with the currently emerging knowledge on IH-triggered immune activation in pregnancies and consequent impacts on offspring. Major inflammatory pathways at play in males, including TLR4/MyD88/NF-κB/MAPK, miRNA/NLRP3, and COX signaling, will be discussed while concerning the therapeutic effects of suppressing specific inflammatory targets within these pathways. Additionally, the effects of IH on immune responses in pregnancies and offspring represent a relatively novel research area and are, consequently, less explored. This review will delve into the limited findings within these understudied populations and discuss the implications for potential new interventions. This may allow for the integration of massive amounts of existing data and further enhance our understanding of this expanding field of research. This, in turn, may springboard the exploration of potential protective mechanisms for treating cardiovascular diseases associated with OSA and the IH it causes, especially in bridging existing knowledge gaps.

## 2. Basic Aspects of IH Exposures and Associated Inflammation

IH, characterized by episodic exposure to hypoxia interspersed with short-term normoxia, is a major component of OSA [13]. Experimentally, IH profiles can be divided into three general exposure categories: (1) acute IH, which involves brief and mild IH exposures (typically above 10% O_2_ for less than 2 h/day) administered during wake cycles for therapeutic purposes [14,15]; (2) chronic IH, which consists of prolonged and severe exposures (usually 5–10% O_2_ and 8 h/day for a few weeks) administered during sleep to simulate OSA conditions; and (3) gestational IH, which models chronic IH exposure during pregnancy to mimic a gestational OSA scenario and to investigate the effects of in utero IH exposures on the offspring [16]. Nevertheless, direct comparisons across studies have been hindered because each laboratory uses a slightly different IH protocol, with diverse magnitudes of hypoxia exposure, number of cycles per hour, and number of hours per day.

IH is a well-recognized trigger of inflammation and oxidative stress, which are closely related during the pathogenesis of cardiovascular complications, especially myocardial infarction, hypertension, and preeclampsia [17,18,19,20]. The reciprocal relationship between inflammation and oxidative stress allows one to easily induce the other. For example, IH generates oxidative stress by decreasing the antioxidant mechanisms and increasing reactive oxygen species (ROS) production during hypoxia/reoxygenation periods [21,22]. The overproduced ROS may directly damage DNA, lipids, and proteins critical for membrane integrity and cellular activity. Imperfect repair of such damage can lead to cell malfunction and apoptosis [23]. Furthermore, ROS may stimulate the transcriptional activities of the pro-inflammatory nuclear factor-kappaB (NF-κB) and activator protein-1 (AP-1) factors, leading to excessive inflammatory cytokine production, adhesion molecule expression, and leukocyte activation [24]. Conversely, inflammation can, in turn, exacerbate oxidative stress [23]. Phagocyte activation undergoes respiratory bursts, during which large amounts of ROS are generated as part of their defense mechanisms against pathogens [25,26]. Further, inflammation can disrupt the normal functioning of mitochondria [27], leading to impaired bioenergetics and excessive ROS production [28,29].

## 3. Chronic IH and Immune Activation in Males

OSA is well-known to elicit systemic inflammation in males [30,31,32], the markers of which correlate with cardiovascular disease in both OSA and non-OSA cohorts [30,32]. In rat models, chronic IH induces aortic tunica media thickening and myocardial injuries, such as cardiac hypertrophy, fibrosis, and apoptosis [33,34,35]. Additionally, chronic IH-induced inflammation causes endothelial dysfunction and injury [36,37], contributing to hypertension and atherosclerosis associated with OSA [38].

### 3.1. TLR4/MyD88/NF-κB/MAPK Signaling

Chronic IH-associated inflammation is suggested to be mediated by Toll-like receptor 4 (TLR4) and NF-κB [33,34,35,39,40,41,42], which are significantly upregulated in apneic patients [43,44,45,46]. TLR4 is a prototypical pattern recognition receptor that senses both infectious stimuli and noninfectious molecules produced as a result of tissue injury and plays an essential role in promoting inflammation [47]. TLR4 requires the adaptor protein, myeloid differentiation factor 88 (MyD88), for effective signaling, leading to the rapid activation of NF-κB and subsequent inflammatory cytokine production [48,49].

In male rats and mice, chronic IH simulating the repeated apnea events of OSA increased TLR4 expression in the heart, liver, and hippocampus [33,39,40]. Chronic IH exposure also significantly increased NF-κB expression in the serum and various tissues such as the aorta, heart, and liver [34,35,39,41,42]. Increased TLR4 and NF-κB levels accompanied the increased expression of inflammatory mediators, including tumor necrosis factor alpha (TNF-α), interleukin (IL)-6, IL-8, IL-1β, monocyte chemoattractant protein-1 (MCP-1), intercellular adhesion molecule-1 (ICAM-1), vascular cell adhesion molecule-1 (VCAM-1), c-reactive protein (CRP), and regulated upon activation normal T cell expressed and secreted (RANTES) [33,34,35,39,40]. The downregulation of TLR4 using either atorvastatin or short hairpin RNA (shRNA) in rats and mice decreased NF-κB expression and attenuated IH-induced oxidative stress, inflammation, and tissue remodeling [33,39,40]. Similarly, NF-κB inhibition via either p50 knockout or IκBα mutant overexpression diminished IH-induced vascular inflammation and injury in mice [41,42].

Mitogen-activated protein kinase (MAPK) is another signaling pathway that can be triggered by TLR4/MyD88. In male rats, chronic IH led to the increased phosphorylation of p38, extracellular signal-regulated kinases (ERKs), and c-Jun N-terminal kinase (JNK) in pancreatic tissue, which were associated with pancreatic inflammation [50,51]. In male mice, chronic IH increased ERK phosphorylation in the liver, contributing to liver fibrosis [52], and increased p38 phosphorylation in brain tissues, contributing to brain injury [53]. A canine model of intermittent airway obstruction caused upregulation of p38 phosphorylation and apoptosis and fibrosis-related factors, which were associated with myocardial apoptosis and fibrosis [54]. Both in vivo [51,53,54] and in vitro [55,56,57,58] experiments showed that suppressing the MAPK pathway partially ameliorated IH-induced inflammation.

MAPK cascades can modulate downstream transcription factors, which ultimately increases the expression of the AP-1 complex [59]. AP-1 regulates critical processes such as proliferation, differentiation, inflammation, and apoptosis [60,61]. IH exposures in vitro induce oxidative stress and subsequently increase c-Fos AP-1 activity in rat PC12 cells [62]. Chronic IH exposures in vivo lead to the increased expression of AP-1 subunits (c-Fos and FosB/ΔFosB), associated with chronic elevations of sympathetic nerve activity and mean arterial pressure in male rats [63,64]. Although AP-1 suppression may be a useful therapeutic approach for treating inflammatory diseases such as asthma, arthritis, and Parkinson’s disease [65], no studies so far have investigated its effects in IH models.

### 3.2. miRNA/NLRP3 Signaling

Exosomes are extracellular vesicles that mediate cell-to-cell communication by delivering DNA, RNA, proteins, or lipids. MicroRNAs (miRNAs) are one of the cargos transported by exosomes and are endogenous non-coding RNAs that regulate gene expression [66]. The biogenesis and functioning of miRNAs can be modulated by oxidative stress [67].

OSA alters exosomal carriers in circulation, especially miRNAs, and promotes endothelial dysfunction [68]. Several in vivo studies have demonstrated the involvement of miRNAs and the nucleotide-binding domain-like receptor protein 3 (NLRP3) inflammasome in chronic IH-induced inflammation and tissue injury [36,69,70,71,72,73,74,75]. During IH exposures, miRNAs such as miR-155 [69], miR-210 [70], and miR-144 [71] are upregulated in renal tissue, endothelium, and serum-derived extracellular vesicles, respectively; the silencing of these miRNAs ameliorate IH-induced inflammation, endothelial dysfunction, and tissue injury [69,70,71].

miRNAs exert post-transcriptional regulation over various genes, including the NLRP3 inflammasome [76]. miR-155, miR-210, and miR-144 all provide positive feedback to NLRP3 activation [77,78,79]. The NLRP3 inflammasome is an oligomeric molecular complex that responds to endogenous stress and triggers innate immune defenses through the maturation of the pro-inflammatory cytokines IL-1β and IL-18 [80].

Clinically, monocytes from individuals with severe OSA present with higher NLRP3 activity than those from control subjects, which directly correlates with the apnea–hypopnea index (AHI) and hypoxemic indices [81]. Monocytes cultured under IH with plasma from healthy humans increase intracellular NLRP3 expression, whereas culturing with plasma from OSA patients increases NLRP3 under both normoxic and IH conditions [81]. The NLRP3 upregulation in OSA patients can be attributed to several potential pathways. For example, NLRP3 overexpression is associated with high levels of oxidized low-density lipoprotein (oxLDL) in plasma from OSA patients with early subclinical atherosclerosis [82], indicating an interaction between dyslipidemia and inflammation in inducing tissue injury. Exposing monocytes from healthy volunteers concomitantly to oxLDL stimulation or plasma from OSA patients with early subclinical atherosclerosis, followed by 16 h of IH, significantly increases NLRP3 activation and IL-1β production, compared to IH exposure alone, suggesting a synergistic act of oxLDL and IH [82]. In addition, several mechanisms of ROS-mediated activation of NLRP3 inflammasome have recently been shown, where apoptosis and mitochondrial damage are potential stimuli leading to NLRP3 inflammasome activation [69,83,84,85,86].

In animal models, chronic IH exposure increases NLRP3 inflammasome expression, and NLRP3 deficiency or inhibition protects against IH-induced inflammation, oxidative stress, and apoptosis in the brain, heart, and vasculature [36,72,73,74,75]. Additionally, in vitro IH exposure fails to induce IL-1β overproduction in bone marrow-derived macrophages isolated from NLRP3 knockout male mice [87], reinforcing the involvement of NLRP3 in IH-induced inflammation.

Notably, NF-κB signaling upregulates miR-155 and miR-210 [88,89], promoting the inflammatory cascade via NLRP3 activation [49], which aggravates myocardial injury and vascular dysfunction [90,91,92]. This reinforces that crosstalk between TLR4/MyD88/NF-κB and miRNA/NLRP3 contributes to chronic IH-induced inflammation and cardiovascular dysfunctions.

Statins, also known as 3-hydroxy-3-methylglutaryl-coenzyme A (HMG-CoA) reductase inhibitors, are a class of cholesterol-lowering medications that also possess cardiovascular protective effects [93]. Atorvastatin, which shows efficacy in attenuating chronic IH-induced myocardial and neural inflammation via suppressing TLR4/MyD88/NF-κB signaling pathways [33,40,94], also suppresses NLRP3 inflammasome activation [95], although this has not been tested in IH models. A recent clinical trial indicates promising protective effects of atorvastatin against cardiovascular risks in OSA patients [96]. However, some studies showed conflicting effects of atorvastatin, where it caused increased p38 phosphorylation, miR-155 upregulation, and NLRP3 activation in mice and rats, leading to inflammation in the adipose tissue and brain [97,98]. Administration of aspirin [98] or rapamycin [97] attenuated the deleterious effects of atorvastatin. This underscores the need for caution when considering atorvastatin usage in the OSA population.

### 3.3. COX-1/Thromboxane and COX-2/PGE_2_ Signaling

Cyclooxygenase 1 (COX-1) and 2 (COX-2) catalyze the rate-limiting step in the production of eicosanoids from arachidonic acid. While COX-1 is constitutively expressed in most tissues and contributes to tissue homeostasis, COX-2 is normally absent but is rapidly inducible at sites of inflammation [99]. COX-catalyzed eicosanoids play fundamental roles in inflammatory response and blood pressure regulation.

OSA subjects presenting with cardiovascular risk factors exhibit elevated urinary 11-dehydrothromboxane B2 (an inactive metabolite of thromboxane A2) compared to OSA subjects free of cardiovascular risk factors and controls [100]. In animal models, chronic IH exposure increased mRNA levels of COX-1 and thromboxane synthase, which correlated with IH-induced atherosclerotic lesion size; the administration of the COX-1 inhibitor SC-560 reduced lesion progression [100]. Additionally, both in vivo [51,101,102,103,104,105] and in vitro [106] studies show that COX-2 is upregulated in response to IH, and there is a concomitant increase in prostaglandin E_2_ (PGE_2_) production. PGE_2_ promotes inflammatory processes and modulates the function of multiple cells involved in the immune response [107].

While aspirin effectively inhibits COX activity and PGE_2_ production, and it is widely used clinically to prevent adverse cardiovascular events, surprisingly, no studies so far have investigated its effects in chronic IH-exposed animal models. This might be because aspirin resistance is prevalent among OSA patients [108]. Moreover, identifying an aspirin dose adequate to prevent inflammation without inducing additional side effects (such as increased bleeding risk) is crucial. A recent cohort study indicated that continuous aspirin use might elevate the incidence of adverse cardiovascular events in hypertensive patients with OSA [109], possibly due to the inhibitory effects of aspirin on platelet aggregation, though the dose of aspirin usage was not specified. Nevertheless, several studies have delved into the therapeutic anti-inflammatory potential of melatonin in mitigating IH-induced tissue injury. It is suggested that melatonin can suppress IH-induced COX-2 overexpression and protect against inflammation and tissue injury in the heart, vasculature, and adrenal medulla [103,104,105]. Melatonin may represent a safer modality than aspirin for suppressing COX-dependent prostaglandin production in OSA.

In addition, flavonoids with antioxidant and anti-inflammation properties may be promising in treating OSA-related cardiovascular diseases, as they target major signaling pathways involved in IH-associated inflammation. For instance, baicalin can reduce ROS production, inhibit p38 MAPK/NF-κB signaling and the NLRP3 inflammasome, and decrease pro-inflammatory cytokines in apolipoprotein E-deficient mice, therefore improving atherosclerosis [110,111]. Chrysoeriol inhibits COX-2 expression and PGE_2_ production without notable cytotoxicity in lipopolysaccharide (LPS)-treated RAW 264.7 cells by ameliorating TLR4/NF-κB, p38 MAPK, and AP-1 activation [112]. A recent study has discovered an inverse relationship between the dietary intake of flavonoids and the risk of sleep disorders [113]. It is worth investigating whether flavonoids show efficacy in IH-induced inflammation and cardiovascular injury.

### 3.4. Shifts in Immune Cell Population and Function

Leukocytes are one of the best-characterized sources of ROS formation and inflammation. Leukocyte accumulation, adhesion, and the initiation of leukocyte/endothelial cell interactions may critically impair endothelial cell function and propagate vascular pathogenesis.

OSA is associated with increased expression of the adhesion molecules MCP-1, CD15 and CD11c by monocytes, increased adherence of monocytes in culture to human endothelial cells and increased intracellular ROS production in some monocyte subpopulations [55,114]. Meanwhile, delayed apoptosis and increased CD15 expression are noted in granulocytes from OSA patients [115], contributing to the prolonged release of inflammatory cytokines and ROS [116,117]. In addition, OSA is associated with a shift in CD4 and CD8 T cells toward type 2 cytokine dominance and increased cytotoxicity [118]. OSA patients exhibit increased expression of natural killer (NK) receptors, CD40 ligand, perforin, and TNF-α in CD8 cytotoxic T lymphocytes [118,119]. Paradoxically, some studies suggest that monocytes from OSA patients exhibit an immunosuppressive phenotype, which includes increased expressions of programmed cell death (PD)-1 receptor and its ligand (PD-L1) [120], a co-inhibitory immune checkpoint to maintain the quiescence of autoreactive T cells [121], reduced CD4 CD8 T cell proliferation and CD8 cytotoxicity [120], and high levels of TGF-β which impairs NK cytotoxicity and maturation [122]. These findings link OSA with cancer incidence and tumor aggressiveness [123]. Alterations of the cellular immune system in OSA have recently been reviewed [124].

There are limited data regarding the effects of chronic IH on immune cell populations and function. In male mice, 4 weeks of IH augmented the macrophage population and ROS release in lung tissue [125]. However, 6 weeks of IH increased PD-L1 expression in splenocytes isolated from male mice, suggestive of an immunosuppressive phenotype, although circulating inflammatory markers were not evaluated [120]. In male rats, IH with a shorter duration (7 days) induced an increase in both circulating M1 (IL-6, TNF-α, IFN-γ, IL-5) and M2 (IL-4, IL-10, IL-13) inflammatory markers [126]. In a different rat model of OSA, 3 h of intermittent airway obstruction significantly increased systemic leukocyte activation and P-selectin expression [127].

Evidenced from in vitro studies, purified granulocytes from healthy humans exposed in vitro to 6 h of IH resulted in increased NF-κB nuclear translocation, p38 MAPK phosphorylation, and expression of IL-8 and its receptor CXCR2 [128]. Exposing purified monocytes from healthy humans to 4.5 h of IH alone had minimal effects on monocyte transcripts, but a combination of IH and IL-1β stimulation together significantly increased chemokine and cytokine gene expression, and led to increased release of MCP-1, IL-6 and TNF-α [129]. Exposing CD14 monocytes isolated from healthy volunteers to alternating preconditioned hypoxic and normoxic media, which mimics IH episodes, resulted in increased TGF-β1 and IL-10 expression, pointing to an NK-suppressing phenotype [122]. Moreover, THP-1 monocytes pre-treated with an inhibitor for either ERK1/2 MAPK or p38 MAPK suppressed the activation of MCP-1 and C-C chemokine receptor 5 (CCR5) expression by IH [55,57]. Using bone marrow-derived macrophages from male mice, IH over 8 h per day for two consecutive days led to a pro-inflammatory M1 phenotype characterized by increased inducible nitric oxide synthase (iNOS) and IL-6 mRNA expression, and a robust increase in NF-κB DNA-binding activity and IL-6 secretion [87]. However, whether these functional alterations are sustained during prolonged IH exposure in vivo remains to be tested.

## 4. Gestational IH and Immune Activation during Pregnancy

The occurrence of OSA increases as pregnancy progresses [3]. While OSA is associated with NF-κB activation, inflammation, oxidative stress, and endothelial dysfunction in a general population [46,130] and OSA is associated with inflammation in women between the ages of 20 and 70 [131], limited research has explored such associations in pregnant women. Recent studies indicate that OSA events positively correlate with systemic inflammation in both normal pregnancies and those with gestational diabetes mellitus (GDM), as indicated by higher circulating levels of TNF-α, IL-1β, IL-8, and IL-10 [132,133]. However, whether the OSA-associated inflammation correlates with compromised cardiovascular function in pregnancy has not yet been established.

OSA is a known risk factor for the development of hypertensive disorders of pregnancy [134,135], including gestational hypertension, preeclampsia, and eclampsia. The severity of OSA positively correlates with the development of gestational hypertension and the severity of preeclampsia [136,137]. Numerous human studies have shown an association between OSA in pregnancy and adverse fetal outcomes commonly seen in preeclampsia, such as intrauterine growth restriction, low Apgar scores, preterm births, and neonatal intensive care unit (NICU) admissions [138].

In murine models, gestational IH exposure serves as a causal factor for maternal development of preeclampsia-like symptoms, including hypertension, proteinuria, uterine artery dysfunction, placental enlargement and morphological change, and fetal growth restriction [139,140,141]. The hypertensive effect of gestational IH is associated with endothelial dysfunction and deficient endothelin relaxation signaling pathways in pregnant dams [142]. Furthermore, dams exposed to gestational IH exhibit increased systemic and placental oxidative stress, along with heightened TNF-α production [140]. However, whether similar inflammatory mechanisms in males and non-pregnant females are also triggered in pregnant females [143] and whether blocking maternal pro-inflammatory molecules can reverse the adverse effects of gestational IH on dam blood pressure, vasculature, and placenta remain to be investigated.

Interestingly, previous studies exposing placental tissue and human umbilical vein endothelial cells (HUVECs) to ischemia/reperfusion (I/R) mimicking placental insufficiency in preeclampsia showed increased oxidative stress and increased production of TNF-α [144,145]. The administration of a TNF-α blocking antibody significantly reduced HUVEC cell activation during I/R [145]. This suggests promising prospects for preventing IH-induced hypertensive disorders of pregnancy by targeting inflammatory signaling.

In addition, miR-155 and miR-210, which are upregulated in IH-exposed male subjects, are also increased in preeclampsia, and serve as predictive biomarkers for preeclampsia development [146,147]. miR-144, however, is downregulated in preeclamptic placentas and plays a protective role by inhibiting pro-apoptotic phosphatase and tensin homolog (PTEN) [148]. The downregulation of miR-144 in preeclampsia aligns with findings from prior in vitro studies using cardiac and intestinal I/R injury models [149,150] but contrasts with observations from the chronic IH model in vivo [71]. It is worth investigating whether gestational IH alters miRNA-mediated inflammation in pregnant females and whether this contributes to hypertensive disorders of pregnancy.

Melatonin has antioxidant, anti-inflammatory, and anti-apoptotic properties [151] and has been shown to exert pleiotropic effects in various endocrinology and cardiology studies [152]. Recent experiments have extensively explored melatonin’s role in pregnancy, and a growing understanding of its physiological functions and its potential therapeutic use to improve maternal and neonatal outcomes has become available [153,154,155]. Melatonin emerges as a new potential candidate in the prevention of pregnancy complications. For example, melatonin supplementation during pregnancy mitigates hypertension and enhances uterine artery endothelial function in hypertensive pregnant mice [156]. Similarly, in rat preeclampsia models, melatonin administration lowers blood pressure and reduces inflammation and oxidative stress in the plasma, placenta, and fetal brain [157,158]. Melatonin supplementation during pregnancy also increases offspring survival during LPS-induced inflammation [159] and reduces blood pressure in the adult offspring [160,161]. However, whether melatonin is protective during gestational IH-induced hypertension and preeclampsia-like symptoms is yet to be investigated. Since melatonin has demonstrated cardiovascular protective effects in chronic IH-exposed males [103,104,105] and hypertensive pregnant females [156,157,158], it is promising that melatonin may exert anti-inflammatory and antioxidant effects also in gestational IH-exposed females, thereby ameliorating adverse cardiovascular events.

## 5. Gestational IH and Offspring Immune Activation

Whether OSA during pregnancy is associated with fetoplacental hypoxia is unclear as there is evidence on both sides. In humans, maternal OSA is reportedly associated with fetal normoblastemia in the placenta, a marker of fetal hypoxia, and with increased placental immunoreactivity of the tissue hypoxia marker, carbonic anhydrase IX [162]. In pregnant mice, 14.5 days of IH exposure during early- to mid-gestation results in higher levels of oxidative stress and hypoxia markers in the placenta [140]. However, contrary evidence from animal models suggests that the fetus is at least partially protected from modest, transient maternal hypoxia [163,164], likely by the placenta. In anesthetized pregnant sheep, real-time partial pressure of oxygen (PO_2_) measurements indicate that a 25 s obstruction results in a ~21 mmHg decrease in maternal blood PO_2_ but only a ~3 mmHg drop in fetal blood [163]. Similarly, pregnant rats exposed to a 5% O_2_ gas mixture have significantly dampened decreases in placental oxygen saturation compared to maternal skin [164]. These data are consistent with findings that hypoxia-inducible factor 1 alpha (HIF-1α) mRNA and its target genes are not altered in placentas or fetal brain at E19 during gestational IH exposure, despite a decline in arterial oxygen saturation in the dam to ~80–85% with each hypoxic episode [165]. Typical oxygen saturation levels in pregnant women with sleep apnea range from 80 to 90% [166,167,168]. Further, the injection of dams with an oxygen-sensing probe (hydroxyprobe) that forms protein thiol adducts in hypoxic cells showed no change in gestational IH-exposed fetal brains relative to controls [165].

Despite the controversy over OSA-associated fetoplacental hypoxia, recent studies consistently highlight adverse fetal and offspring outcomes of gestational OSA. In humans, gestational OSA is associated with low birth weight, accelerated fetal growth, increased adiposity acquisition indicative of potential metabolic disorders, and impaired neurodevelopment in early childhood [169,170,171]. Correspondingly, animal models suggest that gestational IH leads to compromised social and cognitive function [165,172,173], as well as cardiovascular [174,175,176] and metabolic [177,178,179] dysfunctions.

In murine models, gestational IH induced hypertension and endothelial dysfunction, reduced perivascular adiponectin, and elevated TNF-α and oxidative stress marker, 4-hydroxynonenal (4-HNE) in adult male but not female offspring [174,175]. Additionally, gestational IH resulted in tunica intima thickening and the development of pre-atherosclerotic lesions in adult male offspring, which is associated with upregulated NF-κB translocation, p38 MAPK phosphorylation, and the production of TNF-α, IL-8, and CRP [176].

In adolescent male offspring, gestational IH exposure significantly lowered the expression of genes associated with glucose and lipid metabolism and capillary density in respiratory and limb muscles [177]. Impaired mitochondrial metabolism and alteration of oxidative myofibers of the geniohyoid muscle were observed, affecting tongue traction and respiration [178]. Moreover, gestational IH-induced metabolic dysfunction was associated with epigenomic alterations and increased macrophages in visceral white adipose tissue. The macrophage population shifted toward a pro-inflammatory phenotype, as indicated by an increased M1/M2 ratio [179].

The origin of this enduring, sex-specific effect of gestational IH in offspring is unknown, but evidence points to the influence of sex hormones and functional ovaries post-puberty [180]. Evidence suggests that female offspring are hypertensive in the prepubertal age, but after puberty, they become normotensive by 10 weeks of age, coinciding with increased levels of estradiol associated with puberty [180]; in contrast, male offspring are impacted more than their female counterparts in young adulthood, as evidenced by their hypertensive phenotypes [175] and subcortical brain maturation [181]. In addition, the disruption of the neuroendocrine stress pathways may also be a key mechanism by which gestational IH selectively increases disease risk in progeny since neuronal activity in the paraventricular nucleus (PVN) of the hypothalamus, an autonomic control center regulating stress response and blood pressure homeostasis, was specifically increased in male but not female offspring born to gestational IH-exposed dams [182].

The exact mechanisms underlying gestational IH-mediated cardiometabolic dysfunction in offspring, along with the associated changes in immune responses, remain less understood. Further investigations are necessary to elucidate how the effects of gestational IH are transmitted to the offspring and program long-lasting consequences. Interestingly, milk exosomes and their miRNA cargo can pass to the offspring through lactation and may impact the epigenetic programming of various organs and immune responses in the offspring [183], although this has not been tested in gestational IH models. It is worth investigating whether maternal-originated miRNAs contribute to cardiovascular and metabolic diseases in the offspring.

## 6. Conclusions

**Figure 1:**
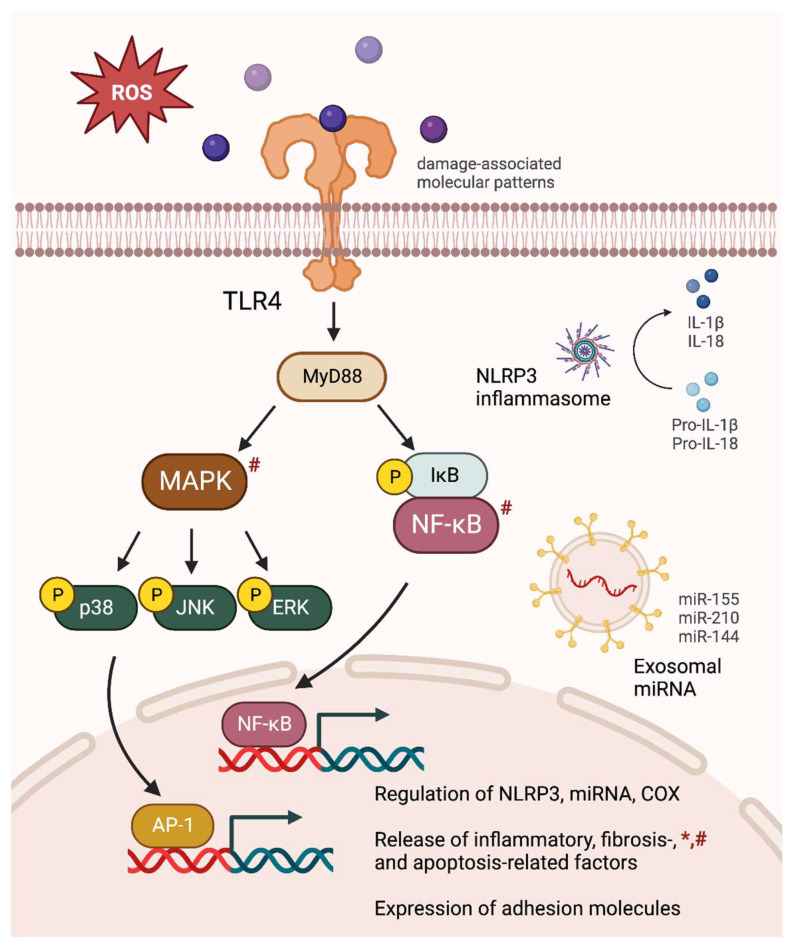
Comprehensive inflammatory consequences of intermittent hypoxia (IH) exposures. IH-induced oxidative stress causes MAPK and NF-κB activation, leading to an upregulation of NLRP3 inflammasome, pro-inflammatory miRNA, and COX signaling. The releases of inflammatory cytokines, fibrosis- and apoptosis-related factors are increased, accompanied by an elevation in adhesion molecule expression. The figure specifically depicts major inflammatory signaling pathways observed in chronic IH-exposed male subjects. Similar alterations found in gestational IH-exposed pregnant females and their offspring are denoted by * and #, respectively.

Inflammation induced by OSA-simulating IH is consistently evident in males, pregnant females, and their offspring and critically contributes to oxidative injury, apoptosis, and cardiovascular dysfunction. In males, the key contributors to chronic IH-induced cardiovascular dysfunction include TLR4/MyD88/NF-κB/MAPK, miRNA/NLRP3, and COX signaling (Figure 1). These pathways are associated with a shift in immune cell populations and function. Specific inhibition of these pathways using either pharmacological methods or transcriptive modifications successfully attenuated inflammation and oxidative stress in the cardiovascular system and improved cardiovascular function. On the other hand, there is a lack of research on the gestational effects of OSA-simulating IH in pregnant moms and their offspring despite the prevalence of OSA during gestation. This highlights the need for future investigations in these understudied populations. Based on the current studies, while it remains uncertain whether the same signaling pathways in males are activated in IH-exposed pregnant females and their offspring, noteworthy similarities have been identified, underscoring the necessity for more in-depth investigations into the underlying inflammatory pathways. More importantly, targeting these inflammatory signaling molecules holds promise for innovative interventions against IH-induced cardiovascular diseases in pregnancies and offspring. Future studies should consider evaluating translatable inflammatory targets in IH-exposed females and offspring.

Nevertheless, it is important to note that inflammation elicited by OSA may result from multiple factors beyond IH per se, such as obesity and nocturnal arousal. For example, 12 weeks of IH increased hepatic TNF-α gene expression only in mice fed a high-cholesterol diet [184]. In OSA patients, CRP levels are significantly correlated with BMI, hip/waist ratio, neck circumference, and frequent arousal [185,186]. This underscores the multifactorial nature of OSA-related inflammation and highlights the importance of considering these contributing factors in understanding and addressing cardiovascular consequences.
